# Beyond ‘migrant background’: how to select relevant, social justice oriented, and feasible social categories in educational research

**DOI:** 10.1007/s10212-022-00611-2

**Published:** 2022-03-11

**Authors:** Jana Vietze, Miriam Schwarzenthal, Ursula Moffitt, Sauro Civitillo

**Affiliations:** 1 Department for Psychology, Education, and Child Studies (DPECS), Erasmus University Rotterdam, P.O. Box 1738, 3000 DR Rotterdam, the Netherlands; 2 Department of Inclusive Education, University of Potsdam, Potsdam, Germany; 3 Department of Psychology, Northwestern University, Evanston, IL USA; 4 Department of Psychology, University of Duisburg-Essen, Essen, Germany

**Keywords:** Migrant background, Labels, Social categories, Discrimination, Academic motivation, National identity

## Abstract

Across continental Europe, educational research samples are often divided by ‘migrant background’, a binary variable criticized for masking participant heterogeneity and reinforcing exclusionary norms of belonging. This study endorses more meaningful, representative, and precise research by offering four guiding questions for selecting relevant, social justice oriented, and feasible social categories for collecting and analysing data in psychological and educational research. Using a preregistered empirical example, we first compare selected social categories (‘migrant background’, family heritage, religion, citizenship, cultural identification, and generation status) in their potential to reveal participant heterogeneity. Second, we investigate differences in means and relations between variables (discrimination experiences, perceived societal Islamophobia, and national identity) and academic motivation among 1335 adolescents in Germany (48% female, *M*_age_ = 14.69). Regression analyses and multigroup SEM revealed differential experiences with and implications of discrimination for academic motivation. Results highlight the need for a deliberate, transparent use of social categories to make discrimination visible and centre participants’ subjective experiences.

Although there is increasing academic engagement with diversity across continental Europe, how best to capture participant heterogeneity related to citizenship, generation status, religion, heritage, and culture poses an ongoing dilemma. ‘Migrant background’ is a binary variable based on whether an individual or their parents or grandparents were born outside the country of residence. In the early 2000s, this term was added to the census in countries including Germany, Austria, and the Scandinavian nations (Simon, 2012; Will, 2019), with the aim of tracking diversifying populations while avoiding the recognition of racial and ethnic categories, which were removed from use after the Holocaust (Möschel, 2011). In the years since, it has become ubiquitous in both popular and research contexts across Europe.

Yet, pushback against ‘migrant background’ has grown in tandem with its use. Part of the critique addresses its lack of theoretical and sociocultural relevance. People with ‘migrant background’ are extremely heterogeneous with regard to salient identity markers such as family heritage, religion, access to formal citizenship, or cultural identification and are grouped solely by their non-native heritage. Even when significant group-based differences are found in research, using ‘migrant background’ without exploring additional identity-relevant factors can reinforce a priori notions of difference without unpacking the myriad structural and individual factors playing into it. Furthermore, ‘migrant background’ is an ascribed category with which many people do not identify (Nesterko & Glaesmer, 2019), meaning, it also lacks personal relevance for research participants.

We situate our study in empirical educational psychology research in Germany, a field that has employed the term ‘migrant background’ inconsistently since the early 2000s (Moffitt & Juang, 2019), including in numerous large-scale national and international educational reports and studies (Will, 2019). Drawing on the critique of ‘migrant background’, we aim to help educational researchers engage in a more deliberate, meaningful, and transparent choice and interpretation of social categories. To do so, we first outline four guiding questions for selecting social categories (see Fig. 1A) based on (1) research questions and aims, (2) theoretical, sociocultural, and participant relevance, (3) social justice implications, and (4) feasibility. Second, we use a preregistered empirical example to compare selected social categories in their potential to (a) highlight adolescent students’ varied experiences, (b) predict mean differences on selected variables, and (c) display differences in relations between selected variables. Finally, we discuss how future researchers can choose and move beyond established social categories, such as ‘migrant background’, to highlight social inequities and promote more meaningful, representative, and precise research (see Fig. 1B).

**Fig. 1:**
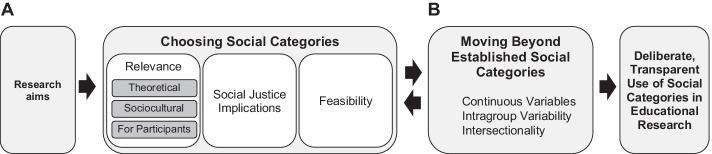
Steps for choosing social categories (section **A**) and suggestions for moving beyond established social categories (section **B**) for a deliberate and transparent use of social categories in educational research

## Guiding question 1: what are the aims of our current research?

When planning a study and before selecting social categories, we as researchers need to clearly define our research questions and aims. Although this guiding question does not presume a specific methodological approach, quantitative scholars are the primary target audience for this paper because categorical social identity constructs are typically employed in survey-based, experimental, or other quantitative research. In our analytic example, we aim to explain differences in academic intrinsic motivation between student groups, a key predictor of school achievement (Taylor et al., 2014), by investigating personal and group-based discrimination experiences (i.e., discrimination experiences and perceived societal Islamophobia) and cultural identification (i.e., national identity), which have each been shown to predict academic outcomes (e.g., Verkuyten et al., 2019). We chose these variables because they allow us to delve into the societal norms and structures upholding multiple forms of inequity, all of which differentially shape the life paths of individuals with ‘migrant background.’

## Guiding question 2: which social categories are relevant for our research aims?

After defining research aims and methodology, we concluded that the current analytic example requires the use of social categories. To identify which social categories may be relevant for our study, we critically reflected on established terminology and thoroughly examined existing literature. To illustrate this process, we briefly review work related to our constructs of interest, namely discrimination experiences, perceived societal Islamophobia, national identity, and their implications for adolescents’ academic motivation in Germany.

### Perceived discrimination and societal Islamophobia

Research based on social identity theory has long argued that discrimination experiences can threaten individual identity needs (e.g., belongingness, esteem, and control) which relate to academic engagement (Civitillo et al., 2021; Verkuyten et al., 2019). In Germany, discrimination based on family heritage and religion is pervasive at both the individual and system levels (SVR-Forschungsbereich, 2018). In Germany’s multi-tiered school system, students with non-German heritage disproportionately attend lower-track schools (Büchler, 2016), a disparity which shapes lifelong academic and professional opportunities. While teachers may not explicitly base their school placement recommendations on children’s ‘migrant background’ (Schneider, 2011), they may hold stereotyped expectations, which leads to judging minority students’ school performance inaccurately and to rating stigmatized student groups (e.g., Turkish heritage students) as less competent and more at fault for errors than their less-stigmatized peers (e.g., Italian heritage students) or German heritage peers (Froehlich et al., 2016; Glock et al., 2015). Thus, discrimination in German schools does not target or affect everyone with ‘migrant background’ in the same way, meaning, there is theoretical and sociocultural relevance for including *family heritage* when examining implications of discrimination experiences in the school context.

Experiencing discrimination from teachers has been linked to lessened school engagement among ethnic minority youth in multiple European settings (Artamonova, 2018; D’hondt et al., 2016). Importantly, anti-immigrant and anti-Muslim sentiment are often intertwined (Reijerse et al., 2013), meaning, Muslim youth face heightened discrimination, including in educational settings (Colak et al., 2020; Welply, 2018). A nationally representative survey (*N* = 2012) in Germany found that nearly half of respondents agreed that, ‘Islam does not belong in Germany’ (Ahrens, 2018), reflecting societal anti-Muslim sentiment and behaviour, also known as Islamophobia (Kunst et al., 2013). Among ethnic minority youth in the USA, awareness of societal stigma has been directly linked to lowered intrinsic motivation (Gillen-O’Neel et al., 2011). Therefore, there is sociocultural relevance for also including *religion* as social category in this study.

### National identity

An exclusionary narrative of national identity is also prevalent in Germany and is reflected in citizenship laws. Although 26% of the population has a ‘migrant background,’ nearly 50% do not have German citizenship (Federal Statistical Office, 2020). Immigration and citizenship laws differ vastly for individuals from within vs. outside the European Union, and who has access to formal citizenship is an ongoing debate informed by racism and anti-Muslim beliefs (Mouritsen, 2013). Some second-generation Turkish-German youth argue that they would still not be accepted as full members of German society even with formal citizenship (Çelik, 2015); other research has linked formal citizenship to national identity among diverse first-generation migrant populations (Maehler et al., 2019). National identity, in turn, has been related to school engagement, though findings remain mixed (Göbel & Preusche, 2019; Schotte et al., 2017). Taken together, this suggests that *citizenship* is also a socioculturally relevant social category when examining national identity and school engagement in Germany.

Although each of these social categories (i.e., family heritage, religion, and citizenship) may also have personal relevance for participants, none captures a self-selected, subjective identity. In the European context, some researchers have used an open response variable assessing participants’ cultural identification and allowing for multiple forms of separate or integrated heritage and national identities. In a German validation study, most migrants identified solely with their heritage country, the second generation reported a mix of hyphenated, solely heritage, and solely German identities, and those of the third generation primarily identified solely as German (Leszczensky & Santiago, 2015). This finding problematizes the common tendency to compare ‘Germans’ (operationalized as individuals without ‘migrant background’) with individuals with ‘migrant background’ (Moffitt & Juang, 2019). Due to the high personal relevance of *generation status* and *cultural identification* for participants’ national identity, we therefore also include these social categories in our study.

## Guiding question 3: what are the social justice implications of our social categories?

Critics have pointed out the negative social justice implications of ‘migrant background’, as its popular use primarily references Muslims and people of colour, reinforcing discriminatory notions of who is ‘German’ and who is ‘Other’ (Elrick & Farah Schwartzman, 2015). In empirical research, by not acknowledging this tendency and pushing against it through conscientious disaggregation, the mechanisms and processes related to population diversity, including power and inequity, remain obfuscated (Helms, 2007). Analogous to work on disaggregating pan-ethnic categories such as Hispanic/Latino or Asian in USA research (e.g., DiPietro & Bursik, 2012), we push for a closer examination of the numerous sources of variability among individuals with ‘migrant background’, which necessitates greater attention to context at each stage of research, including in the planning phases.

It could be argued that any engagement of categorical social identity variables (such as family heritage or religion) further reinforces a priori notions of difference and that even alternatives to ‘migrant background’ only replace one static category with another. A whole body of research has focused on the advantages of renouncing essentialism, i.e., the perception of immutable characteristics underlying groups and individuals (for a review, see Ryazanov & Christenfeld, 2018), especially in educational research (McNess et al., 2015). However, many psychological and educational researchers, including some of the authors, have been inclined to use social categories based on theoretical and methodological considerations and quantitative research traditions. We strongly believe that educational research needs to promote discourses which focus on inequity and diversity rather than on deficit (Aikman et al., 2016) and that this can be mirrored in the selection and use of social categories. Therefore, we encourage all researchers to critically reflect social justice implications as they frame their research questions, select relevant social categories, and draw conclusions based on intergroup differences.

## Guiding question 4: is it feasible to use our social categories?

One reason for the widespread use of ‘migrant background’ is its feasibility, as it was created in continental Europe, where in many countries, it is neither legally possible nor socioculturally meaningful to ask participants about their race or ethnicity. It is not only that race and ethnicity are taboo topics in most of Europe, but many individuals would not know how to answer a question such as, ‘What is your race/ethnicity?’ which is standard in the USA context (e.g., Gyberg et al., 2018). Thus, although we recognize the widespread racism and racialization across the European continent, including racial and ethnic categories in research is neither relevant nor feasible. In our analytic example, we therefore choose *cultural identification* as a means of assessing a socioculturally relevant subjective identity category for academic motivation (e.g., Urdan & Munoz, 2012).

Furthermore, methodological constraints influence which social categories are used. For example, working with existing datasets, the choice of social categories is often limited, as is the case in the current analytic example. Importantly, the nature of ‘migrant background’ as an ascribed, binary category makes it easy to use in quantitative analyses, in which sample size places limits on participant differentiation. If an open-ended item about cultural identification is included in a study with a small sample, the options for making numerous meaningful groups may be limited. Nonetheless, this should not be reason enough to leave out relevant social identity variables that can highlight the heterogeneity within a sample.

## Current analytic example

After careful consideration of relevance, social justice implications, and feasibility, we chose five social categories beyond ‘migrant background’ for empirical investigation in this study: family heritage, religion, generation status, citizenship, and cultural identification.

We preregistered the analytic plan for this research, including study hypotheses (https://osf.io/82r3q/). A complete list of items (https://osf.io/fmvng/), syntaxes (https://osf.io/5a74m/), and Supplemental Tables S1–S6 and Figs. S1–S2 (https://osf.io/4ujpw/) are also available via the OSF. After descriptively exploring the diversity within the group of students with ‘migrant background’, we investigated how using alternative social categories affects mean differences and relations between our variables of interest (i.e., discrimination experiences, perceived societal Islamophobia, national identity, and their implications for intrinsic academic motivation). We tested the following expectations:

- **Hypotheses 1a and 1b**: We expected that more variance can be explained and that group differences become visible when the sample is divided by family heritage and religion (regarding discrimination experiences and perceived societal Islamophobia; Hypothesis 1a) and by citizenship and cultural identification (regarding national identity; Hypothesis 1b) as opposed to by ‘migrant background’.
- **Hypotheses 2a and 2b**: We anticipated that the link between discrimination experiences, perceived societal Islamophobia, and intrinsic motivation differs more when the sample is divided by family heritage and religion (Hypothesis 2a) and between national identity and intrinsic motivation when the sample is divided by citizenship and cultural identification (Hypothesis 2b) as opposed to by ‘migrant background’.

## Method

### Participants and procedure

The study included cross-sectional questionnaire data from 1335 ninth graders (*M*_age_ = 14.69 years, *SD*_age_ = 0.74, 52% male) from 17 secondary schools in Berlin, Germany. The study was part of a larger investigation of cultural diversity norms in secondary schools, as well as teachers’ and students’ intercultural competence and cultural identification (e.g., Civitillo et al., 2019; Schwarzenthal et al., 2020; Vietze et al., 2019). At the time of data collection (i.e., 2016), about one in three secondary school students in Berlin had a so-called migrant background or reported speaking a language other than German at home, placing Berlin at the national average (Senatsverwaltung für Bildung, Jugend und Wissenschaft, 2016; Statistisches Bundesamt, 2017).

All 211 secondary schools in Berlin were contacted by phone and interested school principals received details of the study. Five academic-track and twelve integrated (combined vocational and academic) schools participated. Thus, academic-track schools were slightly underrepresented (29%) compared to the percentage of academic-track schools in Berlin at the time (43%). Participating schools represented a broad range of ethnic compositions (i.e., between 9 and 93% of students with a ‘migrant background’; Senatsverwaltung für Bildung, Jugend und Wissenschaft, 2016). The Berlin Senate Committee for Education, Youth, and Science gave ethics approval for the study. Students received an overview of study goals, learned that participation was voluntary, and that they could withdraw at any time without penalty. Following state regulations, informed consent was not required from parents’ of students over age 14, except on questions related to the parents’ and grandparents’ place of birth. Of the 1335 students in the sample, 52% had a ‘migrant background’ (i.e., at least one parent or grandparent born abroad), 37% did not have a ‘migrant background’, and 11% did not receive consent to provide their parents’ and grandparents’ place of birth. Importantly, the composition of our sample reflected the underrepresentation of students with a ‘migrant background’ in Berlin academic-track compared to integrated schools (Senatsverwaltung für Bildung, Jugend und Wissenschaft, 2016).

### Measures of social categories

#### Migrant background

Participants were asked to report their country of birth, as well as that of their parents and each grandparent. Following the broad definition of ‘migrant background’ (Moffitt & Juang, 2019), we created two groups of students: (1) *without migrant background* (i.e., the participant, parents, and grandparents were born in Germany) and (2) *with migrant background* (i.e., they or at least one parent or grandparent was born outside of Germany).

#### Family heritage

Based on participants’, their parents’, and grandparents’ birth countries, we followed a strict coding procedure (see Supplemental Fig. S1) to group participants into the following family heritage categories: (1) *Germany*, (2) *predominantly Arab countries*, (3) *Turkey*, (4) *Eastern Europe*, (5) *Western Europe* (*other than Germany*), and (6) *other*.

#### Religion

We assessed participants’ religion with the response options (1) Christian (Protestant, Catholic, etc.), (2) Muslim (Sunni, Alevi, etc.), (3) no religion, and (4) another religion (open response).

#### Citizenship

We assessed participants’ formal citizenship with the response options (1) yes, only German citizenship; (2) yes, and citizenship from another country (open response); and (3) no, only citizenship from another country (open response).

#### Cultural identification

We assessed participants’ cultural identification with one item (Leszczensky & Santiago, 2015): ‘Some people consider themselves to be German, for example, others Turkish, and others German-Turkish. What about you? How do you view yourself?’ Response options were (1) *German*, (2) *Turkish*, (3) *German-Turkish*, (4) *Polish*, (5) *German-Polish*, (6) *Russian*, (7) *German-Russian*, *and* (8) s*omething else* (open response). We grouped participants into four groups: (1) *only German identification*, (2) *bicultural identification* (*German and non-German*), (3) *only non-German identification*, and (4) *non-national identification* (*e.g.*, *‘Human’*)*.*

### Measures of variables of interest and covariates

Participants answered questions regarding discrimination experiences, perceived societal Islamophobia, national identity, and intrinsic motivation using 5-point Likert scales from (1) *strongly disagree* to (5) *strongly agree*. We formed mean scores for scales that contained more than one item. Descriptive statistics and correlations between subscales are depicted in Table 1.

**Table 1:** Descriptives and correlations of study variables

	1	2	3	4
1 Discrimination experiences	-	0.28^***^	− 0.11^**^	0.07
2 Perceived societal Islamophobia	0.18^**^	-	− 0.13^***^	− 0.07
3 National identity	− 0.03	0.10*	-	0.19^***^
4 Intrinsic motivation	− 0.04	0.03	0.11^*^	-
*M* (SD) Adolescents without migrant background	1.20 (0.36)	2.73 (0.68)	3.51 (0.86)	2.51 (0.81)
*M* (SD) Adolescents with migrant background	1.68 (0.63)	3.03 (0.81)	3.24 (0.99)	2.65 (0.91)

*N* = 370 adolescents without ‘migrant background’ and *N* = 806 adolescents without ‘migrant background’; ^*^p < 0.05; ^**^p < 0.01; ^***^p < 0.00; correlations for adolescents without ‘migrant background’ below diagonal, correlations for adolescents with ‘migrant background’ above diagonal

#### Discrimination experiences

This 8-item scale (Armenta et al., 2013) includes items assessing personal experiences of ethnic denigration (e.g., ‘Others rejected me because of my heritage culture or the way I look’) and foreigner objectification (e.g., ‘I was asked where I’m from because of the way I look’); α = 0.84. Preliminary analyses indicated that the scale was skewed (skewness = 1.39, kurtosis = 1.69). Thus, we log transformed this variable to improve the skewness levels (skewness = 0.75, kurtosis = 0. − 43).

#### Perceived Islamophobia

We used the Islamophobia scale (Kunst et al., 2013) to assess participants’ perceptions of societal Islamophobia in German society. This scale includes perceptions of general fear (e.g., ‘Many Germans get nervous in the presence of Muslims’), fear of ‘Islamisation’ (e.g., ‘A lot of Germans are afraid that Muslims are going to take over Germany’), and Islamophobia in the media (e.g., ‘German media always presents Muslims as dangerous people’);α = 0.88.

#### National identity

We used the national identity subscale of the German Measure of Youth’s Ethnic and National Identity (Leszczensky & Santiago, 2015) that comprises seven items. Two items address participants’ private regard (e.g., ‘I’m glad to belong to Germany’) and four items assess emotional connectedness to national identity (e.g., ‘I feel closely connected to Germans’). We added one item addressing national belonging (‘I’m proud to belong to Germany’); α = 0.92.

#### Intrinsic motivation

In this 5-item scale (Müller et al., 2007), students were asked about their intrinsic motivation to study and do their schoolwork (e.g., ‘I study and do my schoolwork, because it’s fun for me’); α = 0.90.

#### Covariates

We assessed participants’ gender with the response options (*0*) *male* and (*1*) *female.* As approximation for family socioeconomic status, we assessed the number of books at home, with responses ranging from (*0*) *one or very few* to (*5*) *over 200 books* (Bos et al., 2007).

## Preregistered analytic approach

The full procedure is available via the OSF (https://osf.io/82r3q/) and will be briefly summarized here. After conducting cross-tabulation analyses in SPSS (IBM, 2013), all analyses were run in Mplus (Muthén & Muthén, 1998–2011), using the MLR estimator to deal with violations of normality assumptions and FIML to address missing values. As preliminary analyses, we calculated the intraclass correlations (ICCs) and tested measurement equivalence across all student groupings. To test Hypotheses 1a and 1b, we ran separate multivariate regressions predicting discrimination experiences, perceived Islamophobia, and national identity with dummy-coded social categories (i.e., migrant background, family heritage, religion, citizenship, and cultural identification), controlling for covariates (i.e., gender and the number of books at home). To test Hypotheses 2a and 2b, we ran separate multigroup analyses for each social category (i.e., migrant background and family heritage). In each analysis, after introducing covariates as controls, we introduced discrimination experiences, perceived Islamophobia, and national identity as predictors of intrinsic academic motivation. We then set relations to be equal across social category groups and ran Satorra-Bentler-scaled chi-square difference tests to test whether relations differed between social category groups. If this was the case, we used the model constraint option to test which regression coefficients differed.

## Results and discussion

In the following section, we briefly describe, interpret, and situate the findings of our analytic example in the context of previous research. We then move to a general discussion of how selecting social categories based on the guiding questions we laid out can promote more meaningful, representative, and precise research.

### Cross-tabulation analyses

After establishing ICCs for all constructs and measurement equivalence across social category groups,^1^ cross-tabulation analyses revealed that adolescents with ‘migrant background’ in our sample were indeed very heterogeneous (see Supplemental Table S3). About 80 different heritage countries were represented, with the largest group having Eastern European family heritage (27%), followed by Turkish (21%), Arab (12%), and Western European (12%). About a third of students with ‘migrant background’ were Muslim (36%), closely followed by Christian (28%), and no religion (29%). More than half had only German citizenship (58%), while about a third had dual citizenship (27%) and a minority had only non-German citizenship (12%). Half reported a hyphenated (German and something else) identification (50%), about one third a non-German identification (29%), and a smaller group identified only as German (18%).

### Testing hypotheses 1a and 1b

Results of all regression analyses are reported in the online supplemental material (see Supplemental Table S4). Controlling for gender and number of books at home, students with ‘migrant background’ experienced more discrimination (*β* = 0.34, *p* < 0.001), perceived more Islamophobia (*β* = 0.15, *p* < 0.001), and reported lower national identity (*β* =  − 0.14, *p* < 0.001) than students without ‘migrant background’. First, introducing ‘migrant background’ explained 11% of variance in discrimination experiences and 2% of variance each in perceived Islamophobia and national identity.

Second, we introduced family heritage as a predictor of perceived discrimination and societal Islamophobia. Participants with non-German heritage, across all groups, experienced more discrimination (*β* = 0.08 to 0.33, *p* < 0.01) and perceived more Islamophobia (*β* = 0.07 to 0.17, *p* < 0.05) than those with solely German heritage. These effects were most pronounced in the Arab, Turkish, and ‘other’ heritage groups and less pronounced in the Eastern and Western European heritage groups. Using religion as an alternative predictor led to a similar result. Muslim students experienced more discrimination (*β* = 0.36, *p* < 0.001) and perceived more Islamophobia (*β* = 0.16, *p* < 0.001) than students with no religion, while Christian students did not differ from those with no religion (*β* = 0.03, *p* > 0.05, and *β* =  − 0.03, *p* > 0.05, respectively). The amount of variance explained was slightly higher by family heritage (16% and 4%, respectively) and religion (12% and 3%, respectively) than by ‘migrant background’.

These results support our assumption that important group differences in discrimination experiences and perceived societal Islamophobia are revealed when relevant social-justice oriented social categories are used, though there were small differences in explained variance. In this analytic example, mean differences for discrimination experiences and perceived societal Islamophobia should be treated with caution due to a lack of scalar invariance. Still, the tendency that Arab, Turkish, and ‘other’ heritage groups, as well as Muslim adolescents, may experience the highest levels of discrimination and societal Islamophobia compared to other heritage or religious groups is in line with previous research (e.g., SVR-Forschungsbereich, 2018). Thus, ‘migrant background’ does not adequately capture discrimination based on ethnic or religious group membership (Will, 2019).

Third, we introduced citizenship as a predictor of national identity. Against our assumptions, citizenship neither significantly predicted national identity (for dual citizenship, *β* =  − 0.03, *p* > 0.05; for non-German citizenship, *β* =  − 0.05, *p* > 0.05, compared to only German citizenship) nor explained any variance. Due to the young age of our participants, it may not yet be salient that having German citizenship affects their right to vote and other opportunities for socio-political engagement. Moreover, these participants have not faced the forced choice of maintaining the citizenship of their parents or gaining German citizenship, a policy shaping the experiences of children of migrants from outside the European Union until 2014 (and one which right-wing parties would like to reintroduce; Leubecher, 2018).

Fourth, using cultural identification as an alternative predictor revealed that students who reported a bicultural identification (e.g., ‘Turkish-German’) did not differ from those with monocultural German identification (‘German’) in their national identity (*β* =  − 0.03, *p* > 0.05). However, students who reported a non-German identification (e.g., ‘Turkish’, ‘Turkish-Lebanese’) showed lower national identity than those with a monocultural German identification (*β* =  − 0.35, *p* < 0.001). Cultural identification explained 10% of variance in national identity. To test the robustness of our results, we ran additional analyses without control variables and with log-transformed discrimination experiences as an outcome variable. This led to slightly altered β-coefficients (by max. 0.04), but the pattern of results remained unaffected. Taken together, these findings indicate that cultural identification may be a meaningful social category in mid-adolescence, whereas citizenship may become more meaningful among older participants.

### Testing hypotheses 2a and 2b

**Fig. 2:**
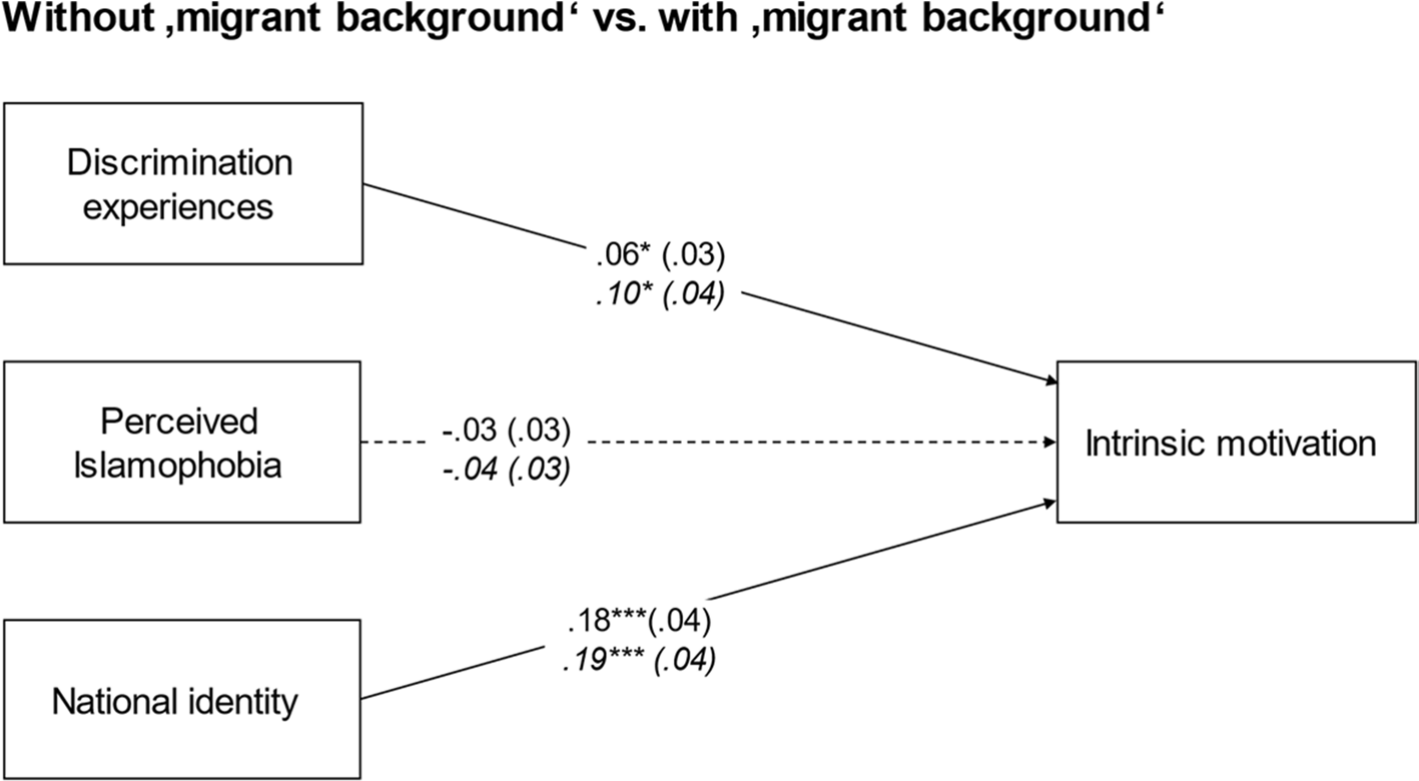
Standardized regression coefficients of multigroup analyses predicting intrinsic motivation with discrimination experiences, perceived Islamophobia, and national identity for students without ‘migrant background’ versus with ‘migrant background (in italics), controlled for gender and family socioeconomic status. Associations were restricted across groups. Standard errors in parentheses; *p < 0.05; ***p < 0.001

**Fig. 3:**
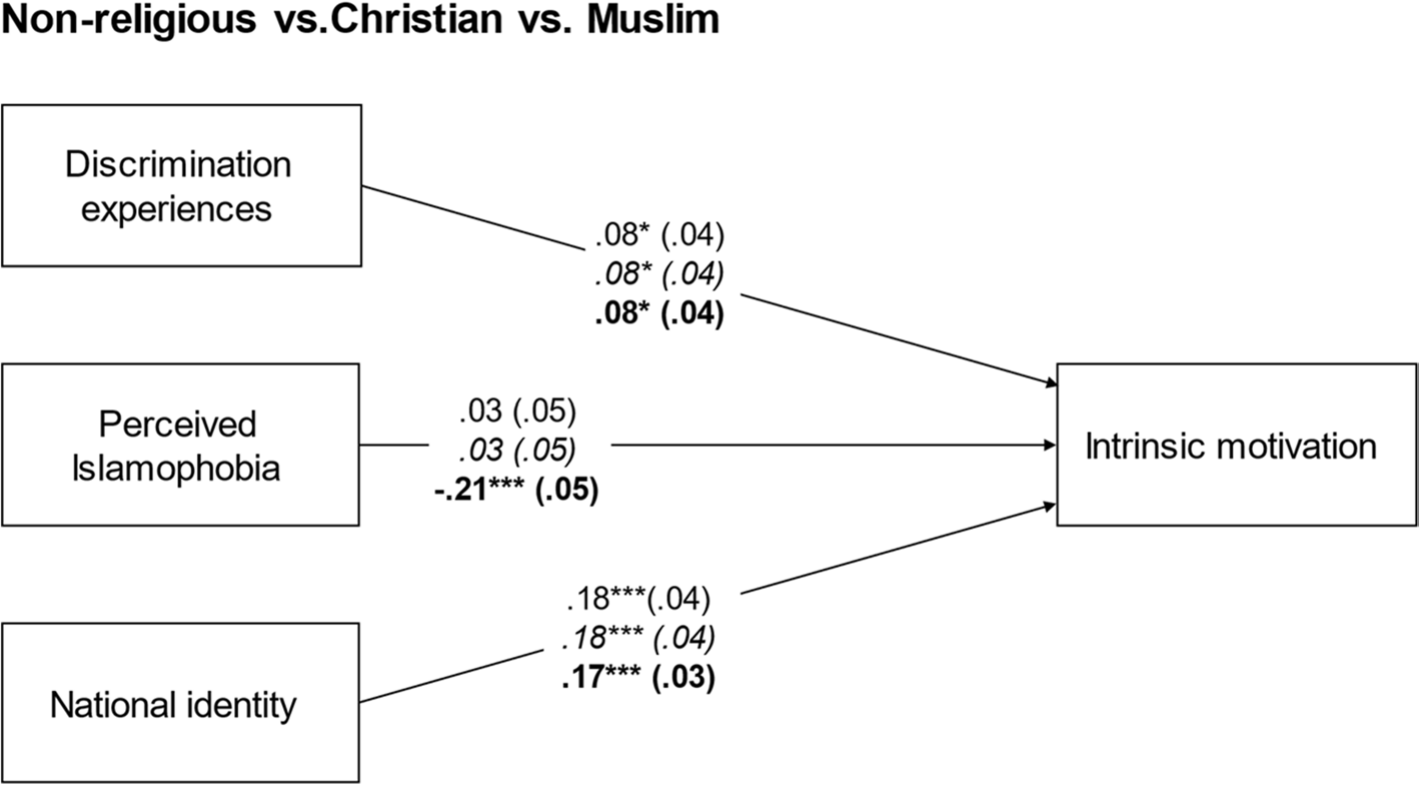
Standardized regression coefficients of multigroup analyses predicting intrinsic motivation with discrimination experiences, perceived Islamophobia, and national identity for students who self-identified as non-religious versus Christian (in italics) versus Muslim (in bold), controlled for gender and family socioeconomic status. Except for the path from perceived Islamophobia to intrinsic motivation, associations were restricted across groups. Standard errors in parentheses; *p < 0.05; ***p < 0.001

We conducted a multigroup regression model with ‘migrant background’ as a grouping category (for model fit indices of all models and coefficients of all final multigroup models, see Supplemental Tables S5 and S6). After restricting relations to be the same across groups, Satorra-Bentler-scaled chi-square difference tests revealed that they did not differ significantly across groups (Δχ_SB_^2^(5) = 3.17, *p* > 0.05; see Fig. 2). Similarly, restricting the paths to be equal across groups did not lead to a significant decrease in model fit when using family heritage (Δχ_SB_^2^(25) = 12.95, *p* > 0.05), citizenship (Δχ_SB_^2^(10) = 5.23, *p* > 0.05), or cultural identification (Δχ_SB_^2^(10) = 2.49, *p* > 0.05) as grouping categories. Thus, relations did not differ significantly across these groups. However, when religion was used as a grouping category, there was a significant decrease in model fit when the paths from predictors to outcomes were restricted to be the same across groups (Δχ_SB_^2^(10) = 13.51, *p* < 0.05; see Fig. 3). Through the model constraint option in Mplus, we found that this was due to the path from perceived Islamophobia to intrinsic motivation, and we accordingly freed this path. In the final model, perceived Islamophobia was only negatively related to intrinsic motivation among Muslim students, while discrimination experiences positively predicted intrinsic motivation in all groups. To check the robustness of our results, additional analyses were run without control variables and with the log-transformed discrimination experiences variable as a predictor, but this did not change the pattern of results.

Based on the knowledge that generation status is a crucial factor when investigating discrimination experiences and their effects (Dimitrova et al., 2017), we ran additional exploratory analyses using generation status as a grouping factor (see Fig. S2). Almost two thirds of students in our sample were second generation (64%), followed by first generation (21%) and third generation (14%). We found that descriptively, the positive relation between discrimination experiences and intrinsic motivation was only present among first-generation youth, non-significant among second-generation youth, and negative among third-generation youth (though the relations did not differ significantly). Similar to findings on bullying victimization (Stevens et al., 2020), first and particularly second-generation youth perceived more societal Islamophobia than those without ‘migrant background’, while this was not the case among the third generation.

The results only partly support our assumption that we would find differential relations between variables when dividing the sample by social categories beyond ‘migrant background’. We found one meaningful difference, namely that Muslim students not only perceived more Islamophobia in German society but were also more affected by these perceptions in their academic motivation. This is an important finding in light of widespread and pervasive anti-Muslim sentiment in Germany (and the whole of continental Europe; Kunst et al., 2013). Yet, overall, our results indicate similar mechanisms across a range of social categories. While assuming homogeneity across social groups (and thus subsuming them under an overarching term such as ‘migrant background’) may be harmful, meaningfully exploring how and when experiences are similar can actually help build solidarity and illuminate commonalities.

The positive relation between discrimination experiences and intrinsic motivation was surprising. In our example, exploring generation status led to the tentative conclusion that compared to their second- and third-generation counterparts, first-generation migrants may anticipate marginalization and are thus not as negatively impacted by it. This finding relates to the so-called immigrant paradox, the phenomenon that first-generation youth show better adaptation outcomes than their peers without personal or family immigration experiences, despite often poorer economic conditions (for a review, see Dimitrova et al., 2017). However, this phenomenon is most likely to occur under specific context conditions, including cultural diversity of the environment, time of residence, and immigrant family reunion (Dimitrova et al., 2016).

## General discussion

The category ‘migrant background’ is commonly used in continental European educational research but has been criticized for being an ascribed, broad category reinforcing inequity. Based on this critique, we proposed that social categories used in quantitative, educational research should be relevant from a theoretical, sociocultural, and participant perspective, be selected to promote social justice, and be feasible for use. We applied these criteria to choose meaningful social categories (i.e., family heritage, religion, citizenship, cultural identification, and generation status) in a preregistered analytic example on the implications of discrimination experiences, perceived societal Islamophobia, and national identity for the academic motivation of secondary school students in Germany.

### What did we gain by going beyond ‘migrant background’?

Our social categories and hypotheses were not exhaustive, and our analytic example did not provide the strong empirical support for our hypotheses we had hoped for. However, by selecting social categories beyond ‘migrant background’ in the current research, our descriptive analyses became more meaningful by *revealing heterogeneity* among adolescents with ‘migrant background’ regarding family heritage, religion, citizenship, cultural identification, and generation status. This variation reflects the different immigration waves in recent German history (Schneider, 2018; Will, 2019). The grandchild of guest workers who immigrated from Italy or Turkey in the 1960s, the child of Polish parents who moved after the opening of national borders in the European Union, and a recent refugee fleeing war-torn Syria would all be classified as having a ‘migrant background’, thereby washing over the differences in their experiences and perspectives. As empirical education and psychology researchers, we wish to highlight the particular importance of disaggregating ‘migrant background’ in work with young people, as not doing so can in fact reinforce the very inequities we research (Moffitt & Juang, 2019; Jugert et al., 2021).

One criticism of the term ‘migrant background’ is that it is too broad to capture discrimination based on phenotypical characteristics or (visible) ethnic or religious group membership (Will, 2019). By using the categories of family heritage and religion in our analyses, we could show that Turkish and Arab heritage as well as Muslim adolescents experienced more discrimination than youth of other heritage or religious groups (SVR-Forschungsbereich, 2018). Moreover, perceived societal Islamophobia was only detrimental for intrinsic academic motivation of Muslim students and not non-Muslim students. One possible reason is that stigmatizing public discourses may be reflected in educational practices, for example in teachers’ negative stereotypes about Turkish-heritage students’ competence and performance (Froehlich et al., 2016). Thus, using these categories made our study results more precise and representative by *highlighting discrimination experiences faced by specific heritage and religious groups* which would have remained hidden if only ‘migrant background’ would have been used.

Our open response option revealed how youth self-identify in terms of their heritage and national identities, countering the false dichotomy often drawn between ‘German’ participants and those with ‘migrant background’ (Moffitt & Juang, 2019). Asking participants about their cultural identification provides an opportunity to take *participants’ own perspective* into account in a context where asking people to report their race or ethnicity is neither relevant nor feasible. Cultural identification not only helped explain a high amount of variance in national identity, it also revealed that identification with one’s heritage culture does not threaten strong national identity, and that the strength and nature of national identity varies across generations. This finding has important implications for research and for the public discourse on the necessity of a German ‘Leitkultur’ (a homogeneous guiding culture regularly called for in discussions of immigration and diversity; Risse, 2018). Importantly, this finding encourages educational institutions not to regard students’ diverse cultural identifications and perspectives as a threat to the social cohesion. Instead, by regarding diversity as a resource, a school climate of cultural pluralism can foster students’ well-being and academic achievement at an individual level (Schachner et al., 2016, 2021), as well as positive intergroup attitudes in the classroom (Schwarzenthal et al., 2018).

### Where do we go from here?

Our analytic example showed the benefits and shortcomings of selecting social categories that are frequently used in educational research on diverse youth in Europe. Increasing academic disparities as the result of the COVID-19 pandemic (Goudeau et al., 2021) highlight the urgency of the social justice concerns in our guiding questions. Thus, we encourage researchers to critically reflect and to move beyond established social categories by considering continuous variables, intragroup variation, and the intersectionality paradigm (see Fig. 1B). Ideally, these considerations should inform decisions around the use of social categories at the beginning of the research process. However, when facing limitations of analysing existing data, as in this study, these considerations should at least guide the interpretation and discussion of results.

To overcome problems inherent in clustering diverse human beings into categories, we as researchers can revise our measures and include more *continuous variables* for assessing individual experiences of social identification and group membership. For example, in an assimilative education system such as in Germany, the *extent* to which youth identify with the national culture has helped explain disparities in academic achievement and school-related values (i.e., importance, utility, and intrinsic values), while including social categories as control variables rather than grouping variables (e.g., Vietze et al., 2019). Using continuous variables allows us to investigate social processes and explore variability in a diverse study population without relying on pre-existing assumptions of difference.

In our attempt to highlight heterogeneity within groups of students with and without ‘migrant background’, in some cases, we found few indications that means or relations differed across groups. This may mean that processes ‘function’ similarly across a range of social groups, but it may also indicate that we were not able to fully capture differential experiences and *intragroup variability within our categories*. For example, the experiences of Turkish heritage individuals may differ depending on their religion or cultural identification, but also with regard to gender or age, over time, and in different relational contexts (Vietze et al., 2018). We encourage researchers to examine variability within any social category, and to explore how it may shape experiences through quantitative approaches, such as longitudinal and moderation analyses, or by drawing on mixed-methods and qualitative approaches to ascertain the relevance of a given social identity construct. Using interviews, focus groups, or open-ended prompts may allow participants to engage in meaning making regarding a given group label (e.g., Bowleg & Bauer, 2016).

Besides investigating variability among subgroups, future research should employ the *paradigm of intersectionality* to illuminate interconnected processes driving social-structural inequalities (Bowleg & Bauer, 2016; Moffitt et al., 2020). For instance, laws limiting religious headscarves specifically impact Muslim women in ways different from how they affect non-Muslim women and Muslim men. Although we found differences between Muslim and non-Muslim participants, examining truly intersectional experiences requires more than simply creating multiplicative variables or interactions and was beyond the scope of this paper. One promising route to address this challenge in future quantitative research is the development of measures that capture experiences at the intersection of multiple social identities and that, for example, do not focus on a single axis of discrimination, such as Islamophobia (for an example, see Scheim & Bauer, 2019).

## Conclusion

The aim of this paper is to encourage researchers to engage in more deliberate and transparent choices regarding and interpretation of social categories in educational research. We believe we have met this aim in three ways: (1) We offered four concrete guiding questions for selecting relevant, social justice oriented, and feasible social categories in line with research aims; (2) we highlighted in a comparative analytical example how the deliberate selection of social categories made our research more meaningful, representative, and precise; and (3) we argued how moving beyond established binary labels, such as ‘migrant background’, can help overcome deficit-oriented research questions and outcomes. We promote future research to challenge prior assumptions of differences and to recognize inequitable societal norms, policies, and structures rather than locating the source of a given ‘problem’ within the population being studied (El-Tayeb, 2014). Importantly, we believe our conclusions can help inform research in related areas, including in studies focusing on acculturation (Juang & Syed, 2019) or gender and sexual identity (Hammack et al., 2021), which have been equally criticized for using dichotomous categories when researching diverse youth populations. A reflective and intentional use of social categories is desirable in the name of both good science and social justice, as it can lead to a more accurate representation of existing diversity, make discrimination and social inequity visible, and respect participants’ own subjective experiences.

## Data Availability

The datasets analysed during the current study are not publicly available but can be made available from the corresponding author on reasonable request. The theoretical background, the hypotheses, and the analytic plan of this study were preregistered on the Open Science framework on October 13, 2019 (https://osf.io/82r3q/). A complete list of items (https://osf.io/fmvng/), syntaxes (https://osf.io/5a74m/), and Supplemental Tables S1–S6 and Figs. S1–S2 (https://osf.io/4ujpw/) are also available via the OSF.

## Footnotes

1 Since the intraclass correlations (ICCs) for all constructs were above 0.05 (see Supplemental Table S1), we accounted for non-independence of observations using the type = complex option in Mplus in all analyses. Scalar measurement equivalence was supported for the national identity and intrinsic motivation scales across social category groups (i.e., migrant background, family heritage, religion, citizenship, and cultural identification). For discrimination experiences and perceived Islamophobia, for some student groupings, only metric equivalence could be established (see Supplemental Table S2). This is in line with earlier findings (Armenta et al., 2013). Thus, relations can be compared across groups, but some of the mean comparisons should be treated with caution.
